# Antiviral Activity of *Haematococcus pluvialis* Algae Extract Is Not Exclusively Due to Astaxanthin

**DOI:** 10.3390/pathogens14080791

**Published:** 2025-08-07

**Authors:** Paula Peinsipp, Tanja Gerlza, Julia Kircher, Kurt Zatloukal, Corinna Jäger, Peter Pucher, Andreas J. Kungl

**Affiliations:** 1 Institute of Pharmaceutical Sciences, University of Graz, Schubertstraße 1, 8010 Graz, Austria; paula.peinsipp@uni-graz.at (P.P.);; 2 BDI-BioLife Science GmbH, Parkring 18, 8074 Raaba-Grambach, Austria; 3 Institute of Pathology, Medical University of Graz, 8010 Graz, Austria

**Keywords:** astaxanthin, algae, antiviral effect, *Haematococcus pluvialis*, SARS-CoV-2

## Abstract

In this study, astaxanthin, which has previously been shown to have antiviral effects, was examined for its dose-dependent potency to inhibit cellular SARS-CoV-2 infections. Naturally occurring astaxanthin is obtained and orally administered as ASX-oleoresin, a composition of different astaxanthin fatty acid esters. We therefore hypothesized that the compound’s beneficial effects are not only related to astaxanthin. Thus, a “green” algae extract (i.e., poor astaxanthin content < 0.2%; ASX^p^) of the microalgae *Haematococcus pluvialis*, as well as an astaxanthin-rich algae extract (astaxanthin content = 20%; ASX^r^), were tested in in vitro cellular viral infection assays. Thereby, it was found that both extracts reduced viral infections significantly. As a potential mode of inhibitory action, the binding of ASX-oleoresin to the viral spike protein was investigated by isothermal fluorescence titration, revealing binding affinities of Kd = 1.05 µM for ASX^r^ and Kd = 1.42 µM for ASX^p^. Based on our data, we conclude that several ASX-oleoresin fractions from *H. pluvialis* exhibit antiviral activity, which extends beyond the known antioxidant activity of astaxanthin. From a molecular dynamic simulation of ASX-oleoresin, fatty acid domains could be considered as activity-chaperoning factors of ASX. Therefore, microalgae biomass should be considered in the future for further antiviral activities.

## 1. Introduction

Astaxanthin, a red-colored lipophilic carotenoid [1], is enjoying increasing popularity as a dietary supplement and cosmetic product [2,3]. Although astaxanthin still has the largest market share in the aquaculture and food industries, where it is used as a coloring agent for salmon and crustaceans [4,5], its strong antioxidant effect leads to a legitimate interest in astaxanthin for human consumption. In addition to its scientifically well-established and frequently demonstrated antioxidant effect, which is reported to be 65 times more potent than vitamin C as an antioxidant [6], astaxanthin has demonstrated anti-inflammatory effects and benefits for skin, ocular, and cardiovascular health [7,8,9].

One of the main sources of natural astaxanthin is the green microalgae *Haematococcus pluvialis* (*H. pluvialis*) [10,11,12]. Although synthetically produced astaxanthin has mainly been used in aquaculture [13], the production of astaxanthin derived from natural sources is increasing worldwide [14]. For human consumption and cosmetics, naturally produced astaxanthin is now exclusively used [14,15].

In *H. pluvialis* and other microorganisms, astaxanthin tends to exist in its esterified form, called oleoresin [16,17]. Astaxanthin can be esterified with one or two fatty acids; in astaxanthin from *H. pluvialis*, monoesters are the most common form of the carotenoid [18].

**Figure 1:**
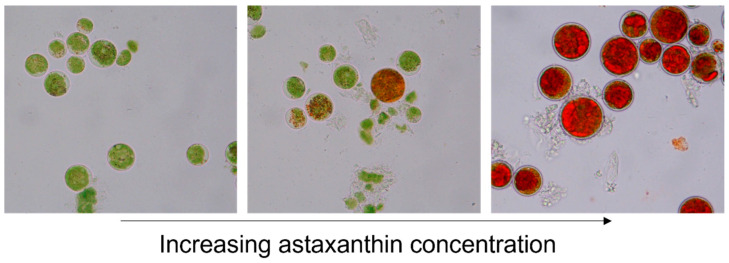
*Haematococcus pluvialis* accumulates red-colored astaxanthin during periods of stress and turns from a green motile cell into a thick-walled, red-colored aplanospore.

The green microalgae *H. pluvialis* is a green, motile, flagellated cell during growth conditions. Upon exposure to stress conditions such as nutrient shortage, increased UV light, or suboptimal salt and pH values, *H. pluvialis* produces astaxanthin and turns from green motile cells into stress-resistant, thick-walled, red-colored aplanospores [1,10,19] (Figure 1).

Besides the above-mentioned beneficial effects on human health, it has also been suggested that astaxanthin has antiviral effects. Talukdar et al. [20] summarized in their review that natural astaxanthin may decrease COVID-19 symptoms by reducing the virus-induced cytokine storm through regulating the expression of pro-inflammatory factors like IL-1β, IL-8, IL-6, or TNF-α [20,21].

However, to the best of our knowledge, there is currently no evidence suggesting that astaxanthin inhibits SARS-CoV-2 infection of host cells. Nevertheless, astaxanthin has been shown to inhibit the entry of other viruses into their host cells. Dona et al. demonstrated that astaxanthin has inhibitory effects on the human papillomavirus L1 protein binding to sperm membranes [22]. An astaxanthin plant extract was also shown to be effective against measles virus infection [23]. Although there are only a few studies dealing with the effect of astaxanthin on HSV-1 infections [23,24], a patent from 2002 claims that astaxanthin is an effective pharmaceutical agent against HSV-1, based on several human case studies [25].

**Figure 2:**
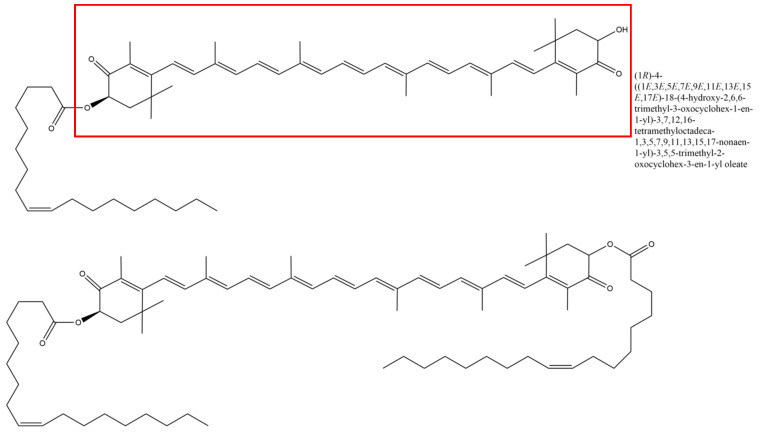
Chemical composition of astaxanthin esterified with one (top panel) or two oleic acid residues. The astaxanthin moiety is encircled in red.

The majority, but not all, of the mentioned studies used natural astaxanthin–oleoresin derived from *H. pluvialis* (see Figure 2). However, the authors still call the active substance “astaxanthin”, even though most of the preparations contained additional substances in the astaxanthin-rich (ASX^r^) algae extract.

This led us to the hypothesis that astaxanthin might not be exclusively responsible for the antiviral effects of algae or plant extracts. Some studies refer to the antimicrobial compounds in *H. pluvialis* extracts, pointing out the difference between the algae’s red and green stage [26]. No such work has yet been published regarding the antiviral potential of *H. pluvialis*. Only Santoyo et al. [10] have focused on a polysaccharide-rich fraction of *H. pluvialis* extracts, showing in their study that a polysaccharide-rich fraction of *H. pluvialis* extracts also inhibit HSV-1 replication without being virucidal itself. Thereby, they suggest that the *H. pluvialis* extract could inhibit HSV-1 infection by interfering with viral attachment to the cell, virus penetration, or virus–host cell fusion. However, it must be taken into consideration that the work lacks information about the astaxanthin content of the polysaccharide-rich *H. pluvialis* extract used in it. This study is in line with numerous other publications attesting to the antiviral effect of marine polysaccharides [27,28]. Moreover, it supports, our statement that astaxanthin is not solely responsible for the antiviral effect of the *H. pluvialis* extract, as is so often assumed in the literature.

In this underlying study, we show for the first time that the antiviral effect of astaxanthin–oleoresin, derived from *H. pluvialis*, is not exclusively due to astaxanthin. To be more precise, we show that within commonly used *H. pluvialis* “astaxanthin” extracts, astaxanthin has an antiviral effect, but it appears not to be the sole active compound in the complex mixture responsible for the antiviral activity. Our studies relate to the inhibition of virus entry into the host cell, virus replication, and/or spreading of SARS-CoV-2. From our data, we are not able to correlate the inhibition of viral infection with further downstream effects, such as excessive immune responses and cytokine storms, which could also be affected by astaxanthin–oleoresin, and should therefore be investigated in the future.

## 2. Materials and Methods

Further information about all methods and results can be obtained from the authors upon request by email.

### 2.1. Production of Haematococcus pluvialis Extracts

The *H. pluvialis* strain used to produce the extracts used in this study was obtained from the BDI-BioLife Science GmbH internal strain collection. In industrial phototrophic production of *H. pluvialis* biomass, cultivation is carried out in two sequential phases. During the initial growth phase, the microalgae proliferate under optimal conditions in bubble column reactors, accumulating biomass without synthesizing astaxanthin. In the subsequent induction phase, the biomass is exposed to stress factors such as nutrient depletion and salt stress in stirred photobioreactors, triggering astaxanthin accumulation. For the production of the red extract (ASX^r^), cells harvested after the induction phase were concentrated by centrifugation with a disk separator, disrupted with a ball mill, dried on an industrial belt dryer, and subjected to supercritical CO_2_ extraction. In contrast, the green extract (ASX^p^) was obtained from biomass that was harvested at the end of the growth phase, dried on a belt dryer without further milling, and subjected to supercritical CO_2_ extraction, resulting in a composition with almost no astaxanthin content. The yield of the ASX^p^ extract was related to the dry biomass by less than 5% (*w*/*w*), with an astaxanthin concentration of <0.2%, while the yield of the ASX^r^ extract is usually around 30% (*w*/*w*). During supercritical CO_2_ extraction of the red extract, the astaxanthin concentration was adjusted to more than 20% by optimizing the extraction parameters.

### 2.2. SARS-CoV-2 Experiment

#### 2.2.1. SARS-CoV-2 Viral Assay

The SARS-CoV-2 omicron strain was propagated in Vero-E6 cells under BSL3 conditions (these cells were chosen since they were shown to contain a very high ACE2 receptor density, which has made them the standard cell line for SARS-CoV-2 studies [29]). Vero-E6 cells were seeded in 48-well plates 24 h before infection at a cell density of 3 × 10^4^ cells per well and incubated at 37 °C and 5% CO_2_ in a serum-free Opti Pro medium. On the day of infection, the virus (MOI 0.002) was preincubated with and without *H. pluvialis* extracts for 60 min in Opti Pro medium.

**Figure 3:**
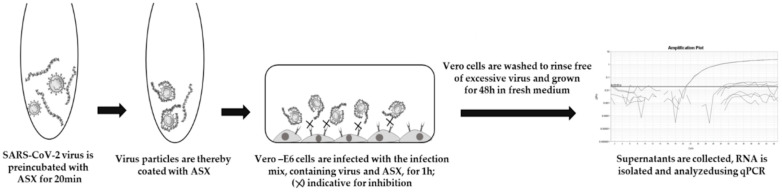
Schematic representation of the experimental set-up. Image adapted from Ennemoser et al. [29]. SARS-CoV-2 virus was pre-incubated with *H. pluvialis* extracts. Vero-E6 cells were infected with the virus/extract mix for 1 h. The cells were washed and incubated for 48 h in the cell culture growth medium. Viral infection rates were determined by quantification of the viral RNA in the supernatant using qPCR.

The viral assay protocol was adapted from Ennemoser et al. [29]. Briefly, as shown in Figure 3, the cells were incubated with an ASX^r^- or an ASX^p^–virus mixture for 1 h at 37 °C and 5% CO_2_. The negative control on the plate with the *H. pluvialis* extracts was contaminated; therefore, non-infected cells from another plate were used as the background for the infection assay. The substance–virus mix was removed after 1 h. After washing the cells twice with 1× PBS (10 mM NaH_2_PO_4_, 137 mM NaCl, pH 7.35), a fresh growth medium with/without *H. pluvialis* extracts in the same concentration was added again. The plate was incubated at 37 °C and 5% CO_2_ for 48 h. Then, the medium was harvested, and SARS-CoV-2 was inactivated using AVL buffer. Cell death/cell toxicity caused by *H. pluvialis* extracts was controlled under the microscope (no additional measures to monitor cytotoxicity beyond microscopic cell density analyses were taken since algae extracts were pre-incubated with the virus, and not with the host cells, which makes induced cell death due to algae extracts rather unlikely).

#### 2.2.2. Viral RNA Quantification

The viral RNA quantification was performed as described previously [29]. In brief, the viral RNA was extracted using the QIAamp Viral RNA Mini Kit according to the manufacturer’s instructions. cDNA synthesis and qPCR were performed in a single step (QuantiTect Probe RT-PCR (Qiagen GmbH, Hilden, Germany)) on a StepOnePlus system (Applied Biosystems/Life Technologies, Carlsbad, CA, USA). The self-designed primers were synthesized at Eurofins Scientific SE (Luxemburg); the sequences can be found in Table 1.

After a 30 min incubation step at 50 °C, the samples were heated to 95 °C for 15 min. For each measurement, 45 cycles (3 s at 95 °C, 30 s at 55 °C) were performed. A positive control was set to 100% infection, while non-infected cells were set to 0% infection. The setting of the threshold line was based on the obtained GAPDH signal, normalizing sample C_T_ values. For amplification and measurement, a StepOnePlus™ Real-Time PCR System Thermal Cycling Block from Applied Biosystems was used in combination with the corresponding StepOne Software V2.3 for data evaluation. The RT-qPCR data was evaluated via the 2^−ΔC^_T_-method, by which the expression level of several target genes is mathematically related to that of an internal control gene (GAPDH). The threshold cycle (C_T_) determines the cycle number, at which the relative fluorescence intensity of a given sample raises significantly above the background signal. The level of significance was indicated by a threshold line, which was adjusted manually to align with the start of the exponential phase.

### 2.3. Isothermal Fluorescence Titration

Isothermal fluorescence titration was performed as described previously [30]. A Fluoromax-4 spectrofluorometer (Horiba, Kyoto, Japan) with an external water bath was used for the titration experiments. A recombinant SARS-CoV-2 P.1 spike protein (tagged with Alexa Fluor^®^ 488, R&D Systems, Minneapolis, MN, USA) at a concentration of 2 nM was equilibrated for 30 min at room temperature. The ligands, i.e., the ASX^r^ or ASX^p^ extracts, respectively, were titrated in steps of 100 µg/mL (12 titration steps; out of a concentrated stock solution to avoid strong dilution) to the protein solution, equilibrated for one min, and then the fluorescence spectra were recorded. The ASX^r^ and ASX^p^ extracts were initially dissolved in 100% DMSO at a concentration of 25 mg/mL. This stock was diluted in 1× PBS to reach a concentration of 2.5 mg/mL. The 2.5 mg/mL stock solution was used to prepare the 100 µg/mL algae extract solutions. DMSO control titration experiments were performed using DMSO as the “ligand” at the same concentrations as in the ASX^r^ or ASX^p^ extracts titrations. DMSO exhibited concentration-dependent fluorescence quenching, which was negligible compared to the effects of ASX^r^ or ASX^p^ extracts. For background correction, the fluorescence spectra from the stepwise titration of the ligands to PBS only were collected and subtracted from the protein fluorescence spectra.

The analysis of the mean values of three independent measurements, plotted against the concentration, was performed according to Gerlza et al. [30] using the Origin (Microcal Inc., Northampton, MA, USA) program. In the following formula, F_i_ represents the initial fluorescence value, while F_max_ denotes the maximum fluorescence. K_d_ is the dissociation constant, and [protein] and [ligand] correspond to the total concentrations of the protein and the GAG ligand, respectively.

$$F=F_{i}+F_{max}\;\left[\frac{K_{d}+\left[protein\right]+\left[ligand\right]-\sqrt{{\left(K_{d}+\left[protein\right]+\left[ligand\right]\right)}^{2}-4\left[protein\right]\left[ligand\right]}}{2\left[protein\right]}\right]$$

### 2.4. ASX-Oleoresin Molecular Modeling

The structure of ASX-oleoresin was generated using the molecule builder tool contained in YASARA [31]. Then, the ASX-oleoresin was energy-minimized with a fixed backbone. The dynamic flexibility of the molecule was evaluated by running a 2 ns MD simulation in an aqueous solution at 298 K and a constant pressure using the AMBER force field. For this purpose, the molecule was put into a rectangular box, which was larger than the molecule along all three axes. The box was filled with TIP3P water. A timestep of 1 fs was chosen for bond, angle, dihedral, and planarity forces, and a timestep of 2 fs was chosen for the intermolecular forces. The cutoff for van der Waals interactions was 7.86 Å, electrostatic forces were calculated without a cutoff using the particle-mesh Ewald algorithm, and bond lengths were not restrained.

### 2.5. Statistical Analyses

For the isothermal fluorescence titrations and Boyden chamber assays, all data are shown as mean + SEM for n observations. All experiments were repeated three times, and statistical analyses were performed via Origin (Microcal Inc., Northampton, MA, USA) or GraphPad v5.04 using Student’s *t*-test. * *p* < 0.05, ** *p* < 0.01 and *** *p* < 0.001 were considered as statistically significant.

## 3. Results

Within this study, we have used astaxanthin-rich *H. pluvialis* extract containing ~20% (*w*/*v*) astaxanthin (termed ASX^r^) and astaxanthin-poor *H. pluvialis* extracts containing < 0.2% (*w*/*v*) astaxanthin (termed ASX^p^). Both samples were extracted from *H. pluvialis* under the same controlled conditions (see Section 2.).

**Figure 4:**
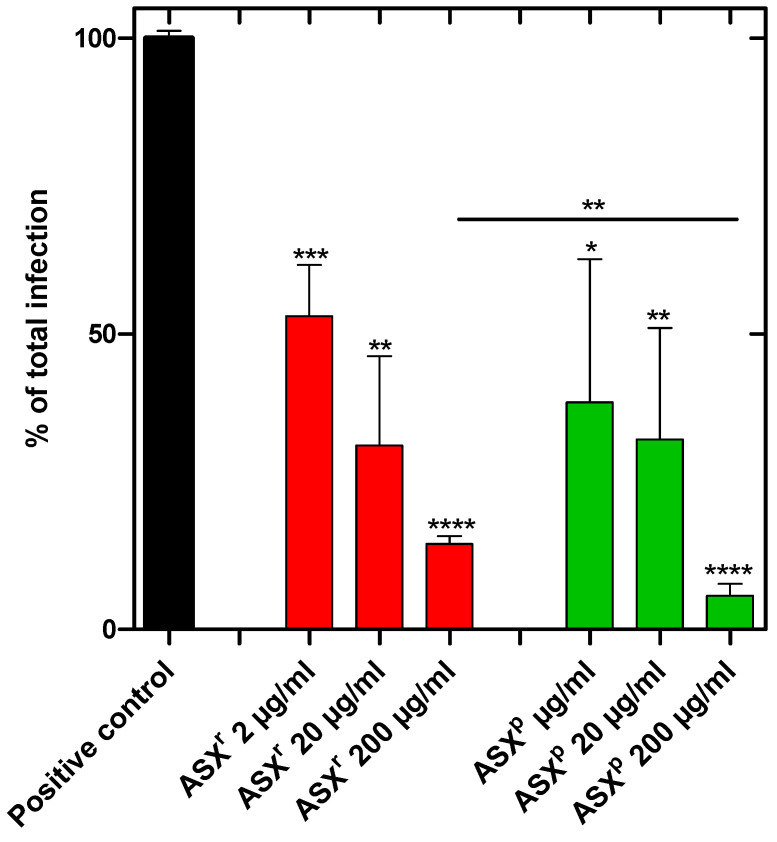
ASX^r^ and ASX^p^ *H. pluvialis* extracts decreased SARS-CoV-2 infection of Vero cells significantly and in a dose-dependent manner. ASX^r^ reduced infection by 85.55%, while ASX^p^ reduced infection by 94.3% in its highest concentration. For statistical analyses, an unpaired *t* test was performed (experiments were recorded in triplicates). *p* values: * < 0.1; ** < 0.01; *** < 0.001; **** < 0.0001. significance.

**Figure 5:**
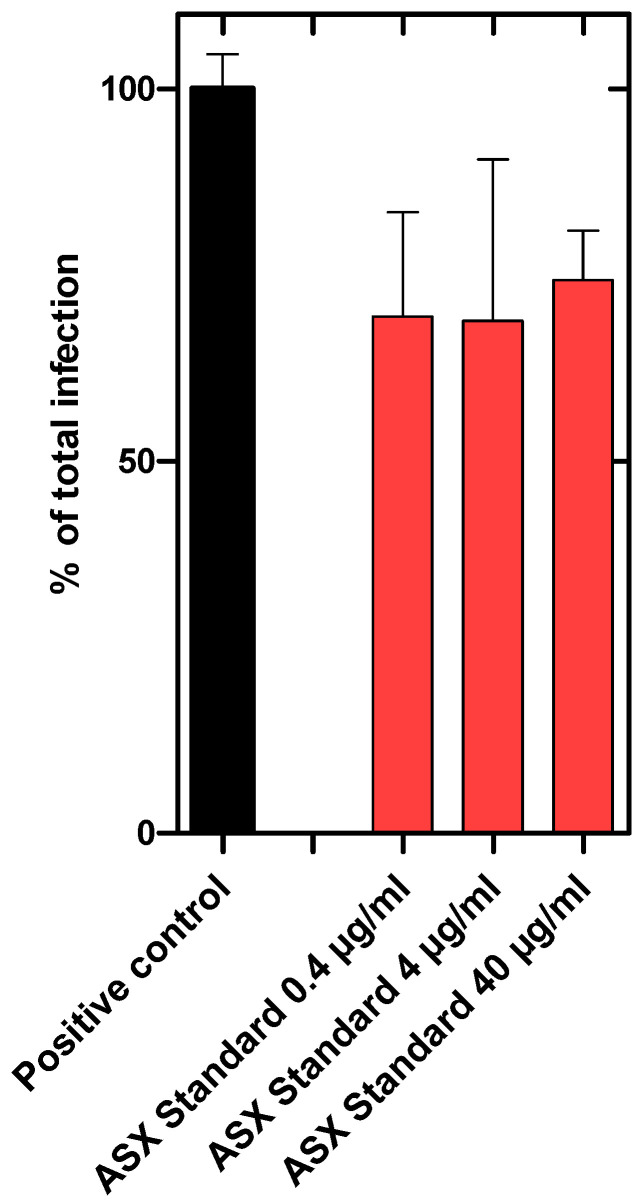
Synthetically produced astaxanthin reduced SARS-CoV-2 infection of Vero cells by 30.6% (0.4 µg/mL), 31.2% (4 µg/mL), and 25.7% (40 µg/mL). The antiviral effect was not dose-dependent. The highest tested astaxanthin standard concentration of 40 µg/mL corresponded to the astaxanthin content (20%) of 200 µg/mL ASX^r^, which is the highest tested concentration of the natural ASX^r^ produced by *H. pluvialis* (experiments were recorded in triplicates).

To investigate the inhibition of viral infection or propagation, Vero-E6 cells were incubated with SARS-CoV-2 (Omicron strain) or SARS-CoV-2 plus algae extract in a BSL3 zone for 1 h. The cells were washed and allowed to grow for another 48 h in the growth medium. Then the cells were harvested, and RNA was prepared to quantify the viral load. We showed that the ASX^r^ and ASX^p^ extracts reduced the infection of the Vero-E6 cells effectively. The infection rate was reduced by ~85% in ASX^r^ [at an inhibitory concentration of 200 µg/mL] treatment, and by ~95% in ASX^p^ treatment. For both extracts, a dose-dependent inhibitory effect was observed. Interestingly, the inhibitory effects of the ASX^r^ and ASX^p^ extracts were found to be similar, and the green extract (ASX^p^) was found to be more effective in a statistically significant way at the highest (200 µg/mL) dose (Figure 4). A synthetically produced astaxanthin standard, however, reduced SARS-CoV-2 infection of Vero cells by only ~30%. Furthermore, there was no dose-dependent effect detected (Figure 5). The maximum concentration tested was 40 µg/mL astaxanthin standard, which corresponds to the astaxanthin content (20%) in the highest ASX^r^ concentration tested of 200 µg/mL.

**Figure 6:**
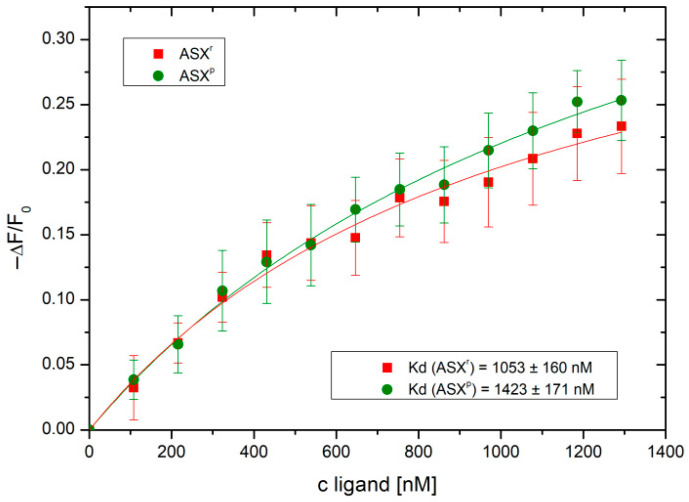
ASX^r^ (red curve) and ASX^p^ (green curve) *H. pluvialis* extracts bind to the SARS-CoV-2 spike protein, as determined by isothermal fluorescence titration (a molecular weight of 928.13 g/mol has been assumed for both astaxanthin fractions, which is derived from the in-process analytics of the extractions; identical amounts of the ASX^r^ and ASX^p^ extracts were used in the experiments).

In order to investigate the potential mode of action by which ASX^r^ and ASX^p^ inhibit SARS-CoV-2 infection/propagation, we studied the interaction of the two compounds with the viral spike protein. For this purpose, isothermal fluorescence studies were performed using an Alexa Fluor^®^ 488-labeled SARS-CoV-2 spike protein. As shown in Figure 6, bi-molecular interaction curves from the IFT experiments gave K_d_ values of 1.05 µM for ASX^r^ and 1.42 µM for ASX^p^. Both *H. pluvialis* extracts were found to bind to the SARS-CoV-2 spike protein, showing a significantly higher affinity for ASX^r^ compared to ASX^p^. No binding was observed for the two extracts and recombinant human ACE2 receptor (see Supplemental Figure S1). Binding of the SARS-CoV-2 spike protein, therefore, seems to be the major mode of the inhibitory activity of the algae extracts. Inhibition of infection was already achieved at 2 µM concentrations of ASX^r^ and ASX^p^ (see Figure 4). This correlates very well with the micromolar K_d_ values found for the binding of the two extracts to the viral spike protein.

**Figure 7:**
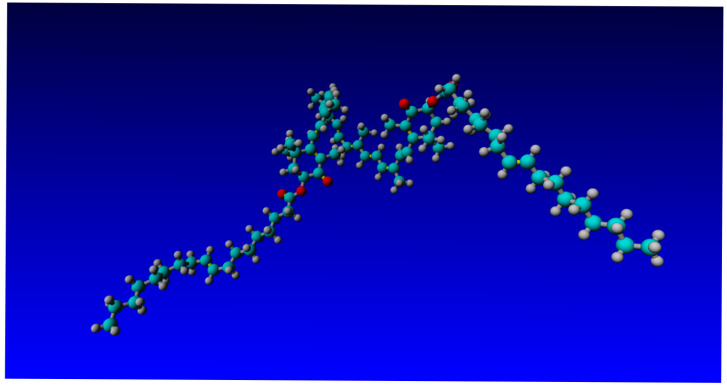
Modeled ASX-oleoresin structure after 1 ns molecular dynamics simulation. Colour code: turquoise, carbon; white, proton; red, oxigen (for more details, see the Section 2).

In order to evaluate the effect of the characteristics of the fatty acid residues on oleoresin, we have performed an in silico analysis of the molecule. We speculated that the fatty acid esters of ASX-oleoresin are responsible (i) for the ASX-amplifying effect (in comparison to non-esterified ASX), as well as (ii) for the directional effect. To be able to correlate these effects with ASX-oleoresin conformation, we performed a short molecular dynamics simulation and structural analysis of the molecule. The molecule adapted an energetically very stable conformation after a 1 ns simulation time (Figure 7). The two fatty acid moieties (both oleic acids) were stretching out at almost 180°, keeping the ASX in the center, almost immobile. The two kinks due to the double bond between C9-C10 of each fatty acid moiety appeared like a molecular hook with which ASX-oleoresin could interact with a lipophilic partner, which resembled a membrane-associated or integral membrane (protein) receptor. Non-esterified ASX would not be able to be targeted in such a manner to a certain (receptor) molecule(s). This is why we postulate that, even at a very low content of ASX, the fatty acid moieties are sufficient to direct the ASX moiety to its receptor, and thus generate the observed high antiviral activity (and also of the ASX^p^). The oleic acids domains could, therefore, be considered as activity-chaperoning factors of ASX.

## 4. Discussion

SARS-CoV-2 is an enveloped (+) RNA virus [32,33] that can progress from mild cold-like symptoms to life-threatening acute respiratory distress syndrome (ARDS) [34]. By 22 December 2024, SARS-CoV-2 had led to over 777 million infections, resulting in more than 7 million deaths [35].

Although the vaccines currently in-use are very effective and the first choice in preventing COVID-19 infections, research in anti-COVID-19 medication is still relevant for those who cannot or do not want to be vaccinated.

Therefore, research on finding new agents to inhibit infection for this virus is not only relevant from a health point of view, but also from a social and economic point of view. The COVID-19 pandemic also showed that the need for broad spectrum antiviral drugs is of great importance in our society. In the future, we will therefore try to investigate the prophylactic antiviral activity building up in people who take ASX-oleoresin as a regular food supplement for longer periods of time. If serum levels are sufficiently high, regular ASX-oleoresin intake could potentially protect and dampen the primary immune response after a first viral encounter. Overactive downstream immune responses (cytokine storm) were the prime cause of SARS-CoV-2 lethal cases. Jimenez & Arias [36] discuss this in their recent article, in what they call the “immunouniverse” of SARS-CoV-2. In the underlying study, we focused on the infection (and indirectly on propagation) of the SARS-CoV-2 virus. Virus-induced downstream cytokine profiles, also affecting the neighboring cells within a tissue, and how this is affected by ASX-oleoresin will be the topic of a future investigation.

The mode of inhibitory action of the ASX-oleoresin extracts is not yet clear. Since RNA was prepared after 48 h of cell growth following infection, we were not able to differentiate between inhibition of viral entry or replication. However, since the virus was pre-treated with ASX^r^ or ASX^p^ for 20 min before cell infection, we believe that both extracts inhibit viral entry by occupying the viral spike protein, rather than replication/propagation. We found, moreover, no clear indication of ASX-oleoresin uptake by the host cells, which is a pre-requisite for the inhibition of viral replication/propagation.

Still, one main point of this study is to focus on the opportunities presented by the total algae extracts, namely the *H. pluvialis* extract, which exceed the known beneficial effects of astaxanthin itself. We have shown that the ASX^r^ and ASX^p^ extracts are, albeit not to the same extent, effective against SARS-CoV-2. The content of the oleoresin extract (as exemplified in the Additional Materials section) is complex, and could contain further bioactive substances. We therefore plan to further fractionate the *H. pluvialis* extract and to test the various fractions for antiviral activity. In addition, it may be assumed that the *H. pluvialis* extract could also lead a reduction in viral infections due to other viruses. Given the proposed mode-of-action (via the virus’ spike protein), it is worthwhile to investigate if the antiviral activity of the *H. pluvialis* extract is restricted to RNA viruses, or if it displays a broader range of anti-infectious activity.

The two different extracts studied here, ASX^r^ and ASX^p^, exhibit interestingly very similar antiviral activity, and consequently, similar binding affinities to the SARS-CoV-2 spike protein. We hypothesize, based on the molecular dynamics simulation of astaxanthin–oleoresin, that the viral protein targeting moiety of ASX^p^ is still astaxanthin, but that the fatty acid esters provide an amplified biological activity. This can also be inferred from the inactivity of the non-esterified synthetic astaxanthin (see Figure 5). Astaxanthin is therefore considered as occupying major binding sites of the viral spike protein, but the fatty acid moieties add to the affinity, as well as to the activity, of the entire molecule by shaping the target in a synergistic, cooperative way. Through this means, not all of the potential binding sites of astaxanthin on its target need to be occupied to obtain full biological activity and high-affinity binding. In a similar way, other components of the algae extracts could synergistically add to the bioactivity of astaxanthin.

Contemplating the pharmacological relevance of our in vitro findings for human therapies, i.e., whether our cellular inhibitory doses could be translated into orally available doses in humans, we saw that the lowest active concentration identified in our in vitro antiviral assays was 2 µg/mL. This concentration cannot be directly translated into achievable serum levels for a human being following oral application. However, Okada et al. [37] have shown in a human study (n = 20) that with a single dose of 48 mg astaxanthin, a C_max_ in the range of 80–150 ng/mL is obtained with a t_max_ in the range of 7.5–21 h. Given the potential pharmacodynamics of the compound (i.e., the active concentration at the site of activity = infection, i.e., neutralizing the viral spike protein), an orally active dose, compared to our in vitro inhibitory active concentrations, seems to be attainable.

In order to utilize the full potential of the *H. pluvialis* extract for the establishment of effective antiviral drugs, the total biomass of the algae *H. pluvialis* should be screened for (further) antiviral substances in the future. The most effective of these could be tested against a broad spectrum of viruses in order to develop a highly effective, natural, broad-spectrum antiviral drug. In addition, based on the knowledge that chemical derivatization of astaxanthin with fatty acids increases the compound’s antiviral activity, we will investigate further chemical modifications of astaxanthin in order to amplify and diversify its full biological potential.

## Figures and Tables

**Table 1:** SARS-CoV-2 qPCR primers. Primer sequences used for SARS-CoV-2 viral RNA quantification. RNAse P was used for RNA quality control.

Gene	FW/RV/Probe	Sequence
N1	Forward	GAC CCC AAA ATC AGC GAAAT
	Reverse	TCT GGT TAC TGC CAG TTG AAT CTG
	Probe	FAM-ACC CCGCAT TAC GTT TGG TGG ACC-BHQ1
RNAse P	Forward	AGA TTT GGA CCT GCG AGC G
	Reverse	GAG CGG CTG TCT CCACAA GT
	Probe	FAM–TTC TGA CCT GAA GGC TCT GCG CG–BHQ-1

## Data Availability

There are no further data publicly available due to the restricted data sharing requested by our industrial partner. Individual requests will, however, be addressed, and fulfilled if possible.

## Supplementary Materials

The following supporting information can be downloaded at: https://www.mdpi.com/article/10.3390/pathogens14080791/s1, Figure S1: Isothermal fluorescence titrations of ASX^r^ (**🟂**) as well as ASX^p^ (**◆**) *H. pluvialis* extracts against Alexa Fluor^®^ 488-labelled human ACE2 protein. No interaction could be observed. Supplemental Material S2: Analysis report of AstaFit® OL100, batch 20000079, from LVA GmbH. The analysis report shows the results of the chemical analysis of ASX^r^ and thus reveals the composition of the substance. The actual composition of ASX^r^ may vary slightly from batch to batch. Supplemental Material S3: Certificate of analysis of the CO_2_ extract of *Haematococcus pluvialis*—oleoresin (ASX^r^) by the company NATECO_2_—Hopfenveredlung St. Johann GmbH, showing the ingredients, heavy metals and microbiology analysis of AstaFit® OL200 (ASXr), batch 20000209.

## Abbreviations

The following abbreviations are used in this manuscript:

ASX	Astaxanthin
ASX^r^	Astaxanthin-rich oleoresin fraction
ASX^p^	Astaxanthin-poor oleoresin fraction
*H. pluvialis*	*Haematococcus pluvialis*
HSV-1	Herpex simplex virus 1
IL-1β	Interleukin-1 beta
IL-6	Interleukin-6
IL-8	Interleukin-8
SARS-CoV-2	Severe acute respiratory syndrome coronavirus 2
TNF-α	Tumor necrosis factor alpha
