# Supplementary material for: Antiviral Activity of Haematococcus pluvialis Algae Extract Is Not Exclusively Due to Astaxanthin
Source: Pathogens. 2025 Aug 7;14(8):791. doi: 10.3390/pathogens14080791 (PMC12389742; doi:10.3390/pathogens14080791)
Supplement: Supplementary file 1 [file pathogens-14-00791-s001.zip › pathogens-3787941 Suppl Material Figure S1.pdf]

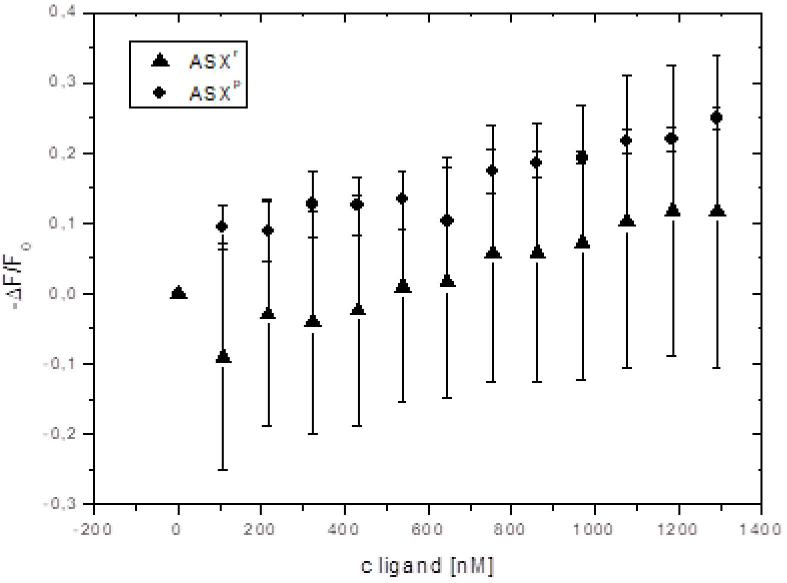


Figure S1: Isothermal fluorescence titrations of $ASX^r$ (▲) as well as $ASX^p$ (◆) *H. pluvialis* extracts against Alexa Fluor® 488-labelled human ACE2 protein. No interaction could be observed.
