# Supplementary material for: Antiviral Activity of Haematococcus pluvialis Algae Extract Is Not Exclusively Due to Astaxanthin
Source: Pathogens. 2025 Aug 7;14(8):791. doi: 10.3390/pathogens14080791 (PMC12389742; doi:10.3390/pathogens14080791)
Supplement: Supplementary file 1 [file pathogens-14-00791-s001.zip › pathogens-3787941 Suppl Material S2.pdf]

LVA
LEBENSMITTEL.
VERTRAUEN.
ANALYSEN.

06.04.2022

Prüfbericht

Auftraggeber BDI - BioLife Science GmbH
Parkring 18
8074 Raaba-Grambach
Österreich

Auftrag **AstaFit® OL100, Batch nr.: 20000079 500 g**
UEB2211466

**Eingangsdatum**	25.03.2022
**Beginn der Prüfung**	25.03.2022
**Ende der Prüfung**	06.04.2022

Probennummer: B2211466

Probenbeschreibung
Überbringungsart: Post
1 Metallbehälter

AstaFit® OL100, Batch nr.: 20000079 500 g;

Chemische Untersuchung

**Natrium (Na)**<br>LVA-UM-IM01; ICP-MS; VE00005716		
Natrium	**<0,001**	g/100 g
**Wasser**<br>LVA-UM-GW01; Gravimetrie; VE00001365		
Wasser	**0,07**	%
**Asche**<br>Gravimetrie; VE00000031		
Asche	**<0,01**	%
**Brennwert**<br>LVA-UM-GN01; Berechnung; VE00000427		
Brennwert	**3544**	kJ/100 g
**Brennwert**<br>LVA-UM-GN01; Berechnung; VE00000354		
Brennwert	**861**	kcal/100 g

Digitally signed by Josef Holzer
Date: 2022.04.06 17:34:19 MESZ

LVA GmbH, Magdeburggasse 10, 3400 Klo[illegible]2-0 | service@lva.at | www.lva.at
Erste Bank: IBAN AT17 2011 1294 6947 4410 | BIC GIBAAT[illegible]1 3236 7000 0030 9351 | BIC RLNWATWW367
DVR: 0722651 | UID Nr. ATU 57127399 | FNr. 236286f | Landesgericht Korneuburg

Akkreditierte Prüfstelle PSID Nr.140 gemäß EN ISO/IEC 17025

Die Prüfergebnisse beziehen sich ausschließlich auf die untersuchte Probe. Eine auszugsweise Vervielfältigung ist ohne unsere schriftliche Genehmigung nicht zulässig.

**Eiweiß**<br>LVA-UM-TE01; Maßanalyse; VE00000154		
Eiweiß (Nx6,25)	**2,0**	%
**Kohlenhydrate**<br>LVA-UM-GN01; Berechnung; VE00000339		
Kohlenhydrate	**5,6**	g/100 g
**Fettgehalt**<br>LVA-UM-GF01; Gravimetrie; VE00005757		
Fett	**92,3**	%
**Gesamtzucker**<br>LVA-UM-HZ01; Berechnung; VE00002919		
Gesamtzucker	**<0,6**	g/100 g
**Fettsäurespektrum**<br>ÖNORM EN ISO 12966-2; GC-FID; VE00000552		
<C6:0 (GC)	**<0,05**	%
Capronsäure, C6:0 (GC)	**<0,05**	%
Caprylsäure, C8:0 (GC)	**<0,05**	%
Caprinsäure, C10:0 (GC)	**<0,05**	%
Undecansäure, C11:0 (GC)	**<0,05**	%
Laurinsäure, C12:0 (GC)	**0,07**	%
Tridecansäure, C13:0 (GC)	**<0,05**	%
Myristinsäure, C14:0 (GC)	**0,51**	%
Myristoleinsäure, C14:1 (GC)	**<0,05**	%
Pentadecansäure, 15:0 (GC)	**<0,05**	%
cis-10-Pentadecensäure, 15:1 (GC)	**<0,05**	%
Palmitinsäure, C16:0 (GC)	**17,5**	%
Palmitoleinsäure, C16:1 (GC)	**0,21**	%
Heptadecansäure, C17:0 (GC)	**0,05**	%
Heptadecensäure, C17:1 (GC)	**<0,05**	%
Stearinsäure, C18:0 (GC)	**0,68**	%
Summe C18:1n4,6,8trans (GC)	**<0,10**	%
Elaidinsäure, C18:1n9trans (GC)	**<0,05**	%
Vaccensäure, C18:1n11trans (GC)	**<0,05**	%
Petroselinsäure, C18:1n6cis (GC)	**<0,05**	%
Ölsäure, C18:1n9cis (GC)	**24,5**	%
cis-Vaccensäure, C18:1n11cis (GC)	**2,9**	%
trans-Linolsäure, C18:2n6trans (GC)	**<0,05**	%
Linolsäure, C18:2n6cis (GC)	**32,0**	%
Arachinsäure, C20:0 (GC)	**0,22**	%
gamma-Linolensäure, C18:3n6 (GC)	**0,94**	%
Eicosensäure, C20:1 (GC)	**0,21**	%
alpha-Linolensäure, C18:3n3 (GC)	**17,2**	%

Akkreditierung Austria 0140 ISO/IEC 17025 T

LVA GmbH, Magdeburggasse 10, 3400 Klosterneuburg | T. +43 2243 26622-0 | service@lva.at | www.lva.at
Erste Bank: IBAN AT17 2011 1294 6947 4410 | BIC GIBAATWWXXX | Raiffeisenbank: IBAN AT31 3236 7000 0030 9351 | BIC RLNWATWW367
DVR: 0722651 | UID Nr. ATU 57127399 | FNr. 236286f | Landesgericht Korneuburg

Akkreditierte Prüfstelle PSID Nr.140 gemäß EN ISO/IEC 17025

Die Prüfergebnisse beziehen sich ausschließlich auf die untersuchte Probe. Eine auszugsweise Vervielfältigung ist ohne unsere schriftliche Genehmigung nicht zulässig.

Heneicosansäure, C21:0 (GC)	**<0,05**	%
Eicosadiensäure, C20:2 (GC)	**0,49**	%
Behensäure, C22:0 (GC)	**0,22**	%
Eicosatriensäure, C20:3n6 (GC)	**0,30**	%
Erucasäure, C22:1n9 (GC)	**<0,05**	%
Eicosatriensäure, C20:3n3 (GC)	**0,08**	%
Arachidonsäure, C20:4n6 (GC)	**<0,05**	%
Tricosansäure, C23:0 (GC)	**1,2**	%
Docosadiensäure, C22:2 (GC)	**<0,05**	%
Eicosapentaensäure, C20:5n3 (GC)	**0,09**	%
Lignocerinsäure, C24:0 (GC)	**0,71**	%
Nervonsäure, C24:1 (GC)	**<0,05**	%
Docosahexaensäure, C22:6n3 (GC)	**<0,05**	%
Summe der trans-Fettsäuren (bezogen auf Fett, ohne Vaccensäure)	**<0,20**	%
Summe der gesättigten Fettsäuren bezogen auf die Gesamtprobe (ber.)	**19,5**	%
Summe der einfach ungesättigten Fettsäuren bezogen auf die Gesamtprobe (ber.)	**25,7**	%
Summe der mehrfach ungesättigten Fettsäuren bezogen auf die Gesamtprobe (ber.)	**47,2**	%
Summe der trans-Fettsäuren bezogen auf die Gesamtprobe (ber.)	**<0,25**	%

Zuckerspektrum
LVA-UM-HZ01; IC; VE00005274

Saccharose	**<0,1**	g/100 g
Glukose	**<0,1**	g/100 g
Fruktose	**<0,1**	g/100 g
Galaktose	**<0,1**	g/100 g
Laktose	**<0,1**	g/100 g
Maltose	**<0,1**	g/100 g

Salz (berechnet aus Natrium)
LVA-UM-IM01; Berechnung; VE00002431

Salz	**<0,003**	g/100 g

LVA GmbH, Magdeburggasse 10, 3400 Klosterneuburg | T. +43 2243 26622-0 | service@lva.at | www.lva.at
Erste Bank: IBAN AT17 2011 1294 6947 4410 | BIC GIBAATWWXXX | Raiffeisenbank: IBAN AT31 3236 7000 0030 9351 | BIC RLNWATWW367
DVR: 0722651 | UID Nr. ATU 57127399 | FNr. 236286f | Landesgericht Korneuburg

Akkreditierte Prüfstelle PSID Nr.140 gemäß EN ISO/IEC 17025

Die Prüfergebnisse beziehen sich ausschließlich auf die untersuchte Probe. Eine auszugsweise Vervielfältigung ist ohne unsere schriftliche Genehmigung nicht zulässig.

LEBENSMITTEL.
VERTRAUEN.
ANALYSEN.

für die Prüfstelle der/die Zeichnungsberechtigte

DI Josef Holzer

KundenbetreuerIn: Renee Eckhardt +43 2243/26622/4202, email: renee.eckhardt@lva.at

Akkreditierung Austria 0140 ISO/IEC 17025 T

LVA GmbH, Magdeburggasse 10, 3400 Klosterneuburg | T. +43 2243 26622-0 | service@lva.at | www.lva.at
Erste Bank: IBAN AT17 2011 1294 6947 4410 | BIC GIBAATWWXXX | Raiffeisenbank: IBAN AT31 3236 7000 0030 9351 | BIC RLNWATWW367
DVR: 0722651 | UID Nr. ATU 57127399 | FNr. 236286f | Landesgericht Korneuburg

Akkreditierte Prüfstelle PSID Nr.140 gemäß EN ISO/IEC 17025

Die Prüfergebnisse beziehen sich ausschließlich auf die untersuchte Probe. Eine auszugsweise Vervielfältigung ist ohne unsere schriftliche Genehmigung nicht zulässig.

LEBENSMITTEL.
VERTRAUEN.
ANALYSEN.

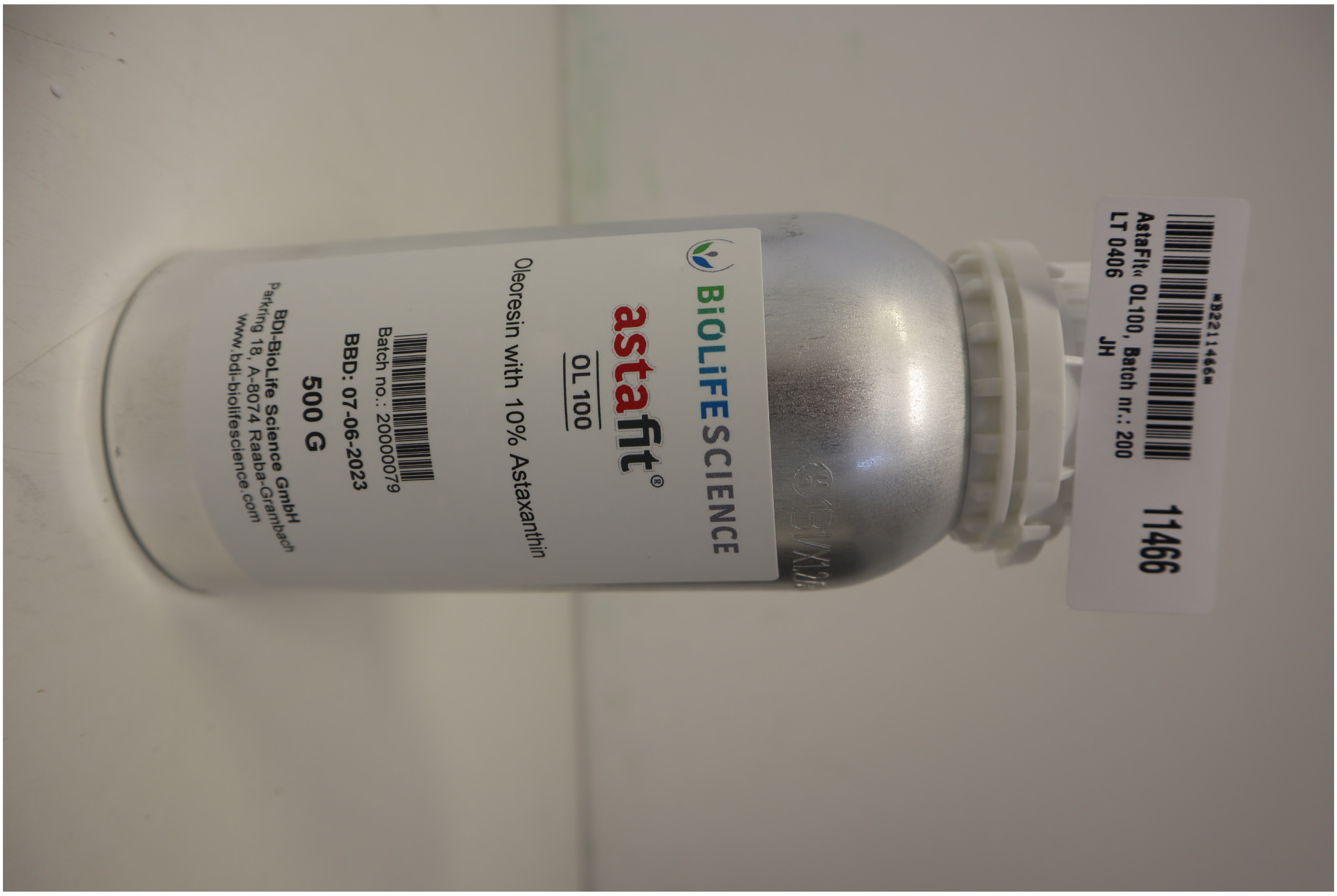

LVA GmbH, Magdeburggasse 10, 3400 Klosterneuburg | T. +43 2243 26622-0 | service@lva.at | www.lva.at
Erste Bank: IBAN AT17 2011 1294 6947 4410 | BIC GIBAATWWXXX | Raiffeisenbank: IBAN AT31 3236 7000 0030 9351 | BIC RLNWATWW367
DVR: 0722651 | UID Nr. ATU 57127399 | FNr. 236286f | Landesgericht Korneuburg

Akkreditierte Prüfstelle PSID Nr.140 gemäß EN ISO/IEC 17025

Die Prüfergebnisse beziehen sich ausschließlich auf die untersuchte Probe. Eine auszugsweise Vervielfältigung ist ohne unsere schriftliche Genehmigung nicht zulässig.
