# Supplementary material for: Antiviral Activity of Haematococcus pluvialis Algae Extract Is Not Exclusively Due to Astaxanthin
Source: Pathogens. 2025 Aug 7;14(8):791. doi: 10.3390/pathogens14080791 (PMC12389742; doi:10.3390/pathogens14080791)
Supplement: Supplementary file 1 [file pathogens-14-00791-s001.zip › pathogens-3787941 Suppl Material S3.pdf]

NATECO$_2$
Extracting the essence of nature

# CERTIFICATE OF ANALYSIS

| | |
|---|---|
| Name of product: | **$CO_2$ Extract of Haematococcus Pluvialis - Oleoresin** |
| Batch number: | 9 BAÖ AstaFitOL200 231018 (Batch -20000209) |
| Production date: | October 20th, 2023 |
| Start / end date of analysis: | October 23th, 2023 |
| Analysis number: | PR2023_3554 |
| Expiration date: | - |
| Description | Dark red viscous oleoresin<br>Characteristic aroma |

**Ingredients:**

| | Method: | Unit | Result Batch: |
|---|---|---|---|
| Total Carotenoid-content | Carotinoide UV Method | [%] | 25,59 |
| Summe Astaxanthin content | HPLC Methode | [%] | **23,60** |
| 13-cis Astaxanthin | | [%] | 3,26 |
| all-trans-Astaxanthin | | [%] | 16,7 |
| 9-cis Astaxanthin | | [%] | 3,59 |
| Lutein | | [%] | 0,185 |
| Canthaxanthin | | [%] | 0,0534 |
| Beta-Carotin | | [%] | 0,168 |

| | | | |
|---|---|---|---|
| Flächenanteile im HPLC-Chromatogramm | Monoester | [%] | 77,5 |
| | Diester | [%] | 22,5 |

**Heavy Metals*:**

| | Method: | Unit | Result Batch: |
|---|---|---|---|
| As (Arsenic) | § 64 LFGB L 00.00-19/1, DIN EN ISO 15763 | mg / kg | - |
| Cd (Cadmium) | § 64 LFGB L 00.00-19/1, DIN EN ISO 15763 | mg / kg | - |
| Pb (Lead) | § 64 LFGB L 00.00-19/1, DIN EN ISO 15763 | mg / kg | - |
| Hg (Mercury) | § 64 LFGB L 00.00-19/1, DIN EN ISO 15763 | mg / kg | - |
| Cr (Chrom) | § 64 LFGB L 00.00-19/1, DIN EN ISO 15763 | mg / kg | - |

**Microbiology**:**

| | Method: | Unit | Result Batch: |
|---|---|---|---|
| Yeasts | ISO 21527-2:2008 | [CFU/g] | - |
| Moulds | ISO 21527-2:2008. | [CFU/g] | - |
| Escherichia coli | DIN EN ISO 16649-3 | [/g] | - |
| Coliforms | ISO 4832:2006-2 | [CFU/g] | - |
| Salmonella sp. | DIN EN ISO 6579 | [/25 g] | - |
| Pseudomonas aeruginosa. | ISO 13720, mod. | [CFU/g] | - |
| Staphylococcus aureus | DIN EN ISO 6888-3 | [CFU/10g] | - |
| Total viable count, aerobic mesophilic 30 °C | ISO 4833-1:2013 | [CFU/g] | - |

**Certificate of analysis does not release customers from their quality responsibility.**

Created on 23.10.2023
*Measured by Eurofins WEJ Contaminants GmbH
**Measured by Eurofins Sofia GmbH

Signed by Product Manager Algae: Dr. Josef Schachtner

i. A Roswitha Walter

Hopfenveredlung St. Johann GmbH
Auenstraße 18-20
85283 Wolnzach | Germany
Telefon +49 8442 66 0 | Fax +49 8442 66 66
contact@nateco2.de | www.nateco2.de

HypoVereinsbank Nürnberg
IBAN-Nr. DE38 7602 0070 0002 0303 65
SWIFT-CODE (BIC): HYVEDEMM460

Sitz: Nürnberg | Registergericht Nürnberg, HR B 33254
Finanzamt Nürnberg | USt-IdNr. DE 133 404 100
Geschäftsführer Oliver Bergner, Dr. Johann Pichlmaier, Florian Herkner
